# Exercise improved bone health in aging mice: a role of SIRT1 in regulating autophagy and osteogenic differentiation of BMSCs

**DOI:** 10.3389/fendo.2023.1156637

**Published:** 2023-07-04

**Authors:** Chenyu Zhu, Haili Ding, Liang Shi, Shihua Zhang, Xiaoyang Tong, Mei Huang, Lifei Liu, Xiaotian Guan, Jun Zou, Yu Yuan, Xi Chen

**Affiliations:** ^1^ School of Kinesiology, Shanghai University of Sport, Shanghai, China; ^2^ School of Sports Science, Wenzhou Medical University, Wenzhou, China; ^3^ Institute of Sports Medicine and Health, Chengdu Sport University, Chengdu, China; ^4^ Department of Gynaecology and Obstetrics, Xinchang People’s Hospital, Shaoxing, China; ^5^ Department of Rehabilitation, The People’s Hospital of Liaoning Province, Shenyang, China; ^6^ School of Exercise and Health, Guangzhou Sport University, Guangzhou, China

**Keywords:** SIRT1, autophagy, exercise, mechanical stretch, bone marrow mesenchymal stem cells, osteoporosis

## Abstract

**Introduction:**

This study was designed to investigate the effect of running exercise on improving bone health in aging mice and explore the role of the SIRT1 in regulating autophagy and osteogenic differentiation of Bone marrow Mesenchymal Stem Cells (BMSCs).

**Methods:**

Twelve-month-old male C57BL/6J mice were used in this study as the aging model and were assigned to treadmill running exercise for eight weeks. Non-exercise male C57BL/6J mice of the same old were used as aging control and five-month-old mice were used as young controls. BMSCs were isolated from mice and subjected to mechanical stretching stimulation in vitro.

**Results:**

The results showed that aging mice had lower bone mass, bone mineral density (BMD), and autophagy than young mice, while running exercise improved BMD and bone mass as well as upregulated autophagy in bone cells. Mechanical loading increased osteogenic differentiation and autophagy in BMSCs, and knockdown of SIRT1 in BMSCs demonstrated that SIRT1-regulated autophagy involved the mechanical loading activation of osteogenic differentiation.

**Conclusion:**

Taken together, this study revealed that exercise improved bone health during aging by activating bone formation, which can be attributed to osteogenic differentiation of BMSCs through the activation of SIRT1-mediated autophagy. The mechanisms underlying this effect may involve mechanical loading.

## 1 Introduction

Osteoporosis is a common systemic disease of bone metabolism characterized by decreased bone mass and mineral density, impaired bone microarchitecture, and then leading to increased bone fragility and fracture susceptibility (1). Several factors that contribute to the processing of osteoporosis, senescence, and estrogen deficiency play significant roles (2). Senile osteoporosis (SOP) is defined as age-related bone mass loss or impaired structure in the skeletal system, which typically begins around 50 years of age and accelerates significantly after the age of 70 (3, 4). The function of differentiation potential and osteogenesis in bone marrow mesenchymal stem cells (BMSCs) during aging is critical for SOP (5, 6). It has been shown that reducing the differentiation of BMSCs into osteoblasts and increasing their differentiation into adipocytes lead to diminished bone formation and results in SOP (7). Increasing evidence suggests that appropriate exercise promotes bone formation and improves bone mass, thereby preventing osteoporosis. Exercise-induced mechanical loading increases the osteogenic differentiation of BMSCs, promotes bone formation, inhibits the abnormal activity of osteoclasts, and attenuates bone resorption by interacting with hormones, cytokines, and signaling pathways (8, 9). Conversely, lack of exercise leads to loss of bone mass, which is associated with increased adipogenic differentiation in BMSC (10). Wu et al. found that tensile stimulation promoted the expression of ion channel proteins Piezo1, Piezo2, and transient receptor potential vanilloid 4 in BMSC, mediating Ca^2+^ inward flow and promoting osteogenic differentiation (11, 12). In addition to the classical mechanosensitive proteins, Sirtuin 1 (SIRT1) has been identified as a novel mechanosensitive factor in BMSC. It has been shown that mechanical stretch with 5% intensity promotes SIRT1 expression in BMSCs, leading to improved antioxidant capacity and promoting osteogenic differentiation (13). Autophagy also plays a crucial role in response to mechanical stimulation, and its activation is essential for promoting osteogenic differentiation. Hind limb unloading resulted in massive bone loss in mice, accompanied by reduced autophagic activation of osteoblasts. However, activation of autophagy significantly increased osteoblast differentiation and reduced bone loss induced due to unloading (14).

SIRT1 is a nicotinamide adenine dinucleotide–dependent histone deacetylase, which is involved in the regulation of various cellular physiopathological processes, such as cell proliferation, apoptosis, senescence, oxidative stress, and autophagy (15). It was found that, in BMSC-specific SIRT1 knockout mice, both trabeculae and cortex were significantly reduced, whereas treatment with the SIRT1 agonist restored bone mineral density (BMD) in tibiae and reduced bone marrow adipogenesis, indicating that SIRT1 upregulates osteogenic differentiation and downregulates adipogenic differentiation in BMSCs (16). Further research revealed that the SIRT1 agonist resveratrol rescued the inhibition of osteogenic differentiation by premature senescence and simultaneously delayed BMSC senescence through the reduction of senescence-related markers such as p16INK4α, p21, p16, and p38; preserved more functionally normal mature osteoblasts; and promoted osteoid mineralization (17, 18). In addition, SIRT1 inhibits oxidative stress in BMSCs by deacetylating Forkhead box O3 (FOXO3a), thereby reducing osteoblast senescence and enhancing osteogenic differentiation, leading to the alleviation of osteoporosis (19).

Autophagy is a crucial metabolic process that helps cells survive under conditions of nutrient or energy deprivation, infection, oxidative stress, or hypoxia (20). The cell engulfs its cytoplasm or organelles into vesicles, forming autophagosomes, and is transported to the lysosome for degradation. This process removes damaged or aging organelles and preserves basic cellular stability (21). Autophagy has been extensively studied as a mechanism for anti-aging agents and the alleviation of age-related diseases (22). Autophagy is necessary for maintaining stem cell stemness and differentiation capacity and is an important mechanism for maintaining the youthful state of stem cells, and inhibition of autophagy leads to cellular senescence (23). Osteoporosis is reported to be accompanied by high levels of oxidative stress, whereas activation of autophagy protects BMSCs from oxidative stress, thereby counteracting the aging effects of oxidative stress on BMSCs and promoting osteogenic differentiation (24). Yang et al. demonstrated that SIRT1 improves bone mass in osteoporotic rats by activating the signaling pathway of PI3K/AKT/mTOR to activate autophagy in osteoblasts (25).

On the basis of preliminary studies, SIRT1 is a mechanosensitive protein in BMSCs in response to mechanical stimulation and may regulate the activation of autophagy, which is essential in bone formation. However, it is unclear whether exercise or mechanical stimulation regulates SIRT1-mediated autophagy in BMSCs. Thus, this study was designed to investigate the effect of running exercise on improving bone health in aging mice and explore the role of the SIRT1 in regulating autophagy and osteogenic differentiation of BMSCs.

## 2 Materials and methods

The animal study was approved by the Ethical Committee of the Shanghai University of Sport and was carried out strictly under Chinese law and local ethics committees (102772022DW010).

### 2.1 Animals

Twelve-month-old male C57BL/6J mice were randomly divided into an aging control group (A, n = 10) and an exercise group (E, n = 10), and 5-month-old male C57BL/6J mice were recruited as the young control group (Y, n = 10). During the experiments, all mice were raised under specific pathogen–free conditions.

### 2.2 Running exercise intervention and the tissue prepared

**Figure 1:**
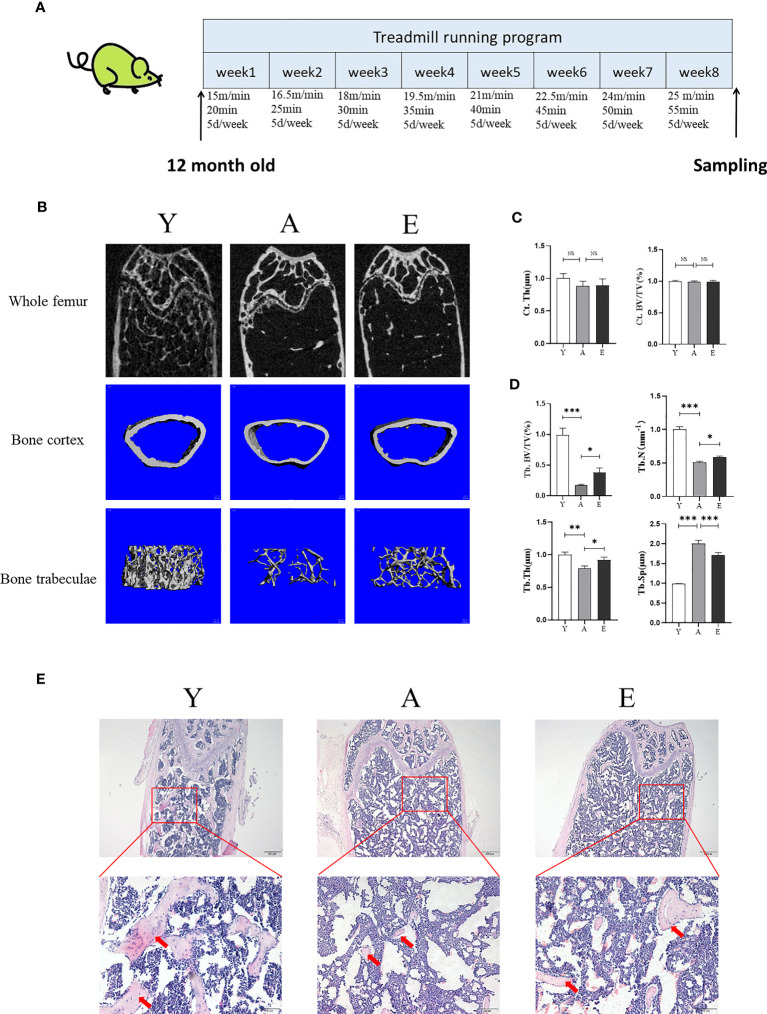
Exercise improves bone mass in aging mice. **(A)** The diagram of animal experiment. **(B)** Representative 2D and 3D micro-CT images of mice fumer. **(C, D)** Quantitative analysis of bone structural parameters: Ct.Th, cortical bone; Ct.BV/TV, cortical bone volume; Tb.BV/TV, trabecular bone volume; Tb.N, trabecular number; Tb.Th, trabecular thickness; Tb.Sp, trabecular separation. **(E)** Representative images of H&E stained decalcified femoral sections. Scale bars, 200 and 50 µm. Red arrow, trabecular bone. Y, young control group; A, aging control group; E, exercise group. *P < 0.05, **P < 0.01, and ***P < 0.001; NS, no significant difference.

Groups Y and A were fed statically without any intervention. Group E performed treadmill running exercise intervention (8 weeks, 5 days/week, 1 time/day). A gradually increasing intensity running program was used, which corresponded to 60%~80% peak workload (26, 27). In brief, the running speed and duration of the first week were 15m/min and 20 min; from the second to the eighth week, the speed increased by 1.5m/min per week until 25m/min, and the duration increased by 5 min per week. The slope of the running platform was 0° throughout the training process (**Figure 1**).

The mice were euthanized when the treadmill running intervention was completed, and the tibias and femurs were dissected carefully without soft tissue for further experiments.

### 2.3 Micro–computed tomography and histomorphometry

The femur were fixed in 4% paraformaldehyde for 1 week and placed in a universal L145 scaffold system, and the samples were fixed using polyethylene foam to avoid displacement. After localization, scans were performed using micro–computed tomography (micro-CT) (scanco medical vivaCT80). The scanning parameters were as follows: working voltage, 55 kVp; current, 145 μA; power, 8 W; scanning mode, continuous ROT; scanning layer thickness, 9 μm; FOV radius, 31.0 mm; exposure time, 250 ms; resolution, native; number of scanning layers per segment, 388 layers; and BH calibration, 1,200 mgHA/cc. The area 1 mm away from the femoral growth plate was selected as the area of interest for 3D image reconstruction and cancellous bone morphological analysis, and the cortical volume fraction (Ct.BV/TV), cortical area fraction (Ct.Ar/Tt.Ar), trabecular volume (Tb.BV/TV), trabecular thickness (Tb.Th), trabecular number (Tb.N), and trabecular separation (Tb.Sp) were automatically calculated.

After the micro-CT analysis, all femurs were decalcified in 10% ethylene diamine tetraacetie acid, decalcifying solution at room temperature for 30 days; then, the samples were processed into wax by using ethanol and xylene, embedded in paraffin blocks, and sliced into 5-µm sections using a microtome. Hematoxylin and eosin (H&E) and immunofluorescence (IF) staining were performed.

### 2.4 Isolation and culture of BMSCs

Four-week-old male C57BL/6J mice were used for BMSC isolation. The bone marrow was obtained by sterilizing mouse femurs and tibias and by flushing them to isolate the cells and then was suspended in culture medium [α–minimum essential medium (α-MEM) supplemented with 10% fetal bovine serum] in a humidified incubator maintained. After 3 days, the non-adherent cells were removed, and the culture medium was changed, with subsequent changes every 2 days. The cells were passaged and used in this study.

### 2.5 Application of transfection and mechanical stretch to BMSCs

The second generation of BMSCs was seeded in BioFlex six-well plates at a density of 1 × 10^5^ cells/ml. When the cells reached 70% confluence, the medium was replaced with α-MEM basic medium without serum, penicillin, and streptomycin. Then, the prepared Small interfering RNA (siRNA) (siRNA-SIRT1, siRNA-NC, 200 pmol per well) (GenePharma)–lipo3000 (Invitrogen, 7.5 μl per well) transfection complexes were added to the corresponding well, gently shaken, and mixed into the cell culture incubator. After incubating for 6–8 h, the cells were changed to osteogenic-induced differentiation medium and then subjected to mechanical stretch intervention for 7 days or 1 day. According to our previous studies, the stretch intervention protocol was set as follows: 4% deformation intensity, 0.5 Hz, 4 h. The 7-day groups were subjected to mechanical stretch every 2 days (28, 29). The negative control cells were cultured in BioFlex plates without stretch intervention.

### 2.6 RNA isolation and real-time PCR

Total RNA was extracted from femurs and BMSCs using TRIzol reagent (Invitrogen) with the A260/280 from 1.93 to 2.05 (30). The Reverse Transcription Reagent Kit (Takara RR037A) was used to obtain cDNA. The reaction mixture, consisting of reagents, primers (**Table 1**), and samples, was assembled in a PCR plate using the SYBR® Green Pro Taq HS Premix qPCR Kit (Rox AG11718). The mRNA expression of the target genes was determined using 2^−ΔΔt^, with β-actin and GAPDH serving as an internal reference.

**Table 1:** The list primers.

Gene	5′-3′ Primer Sequence
GAPDH-F GAPDH-R	ACTCCACTCACGGCAAATTC TCTCCATGGTGGTGAAGACA
β-actin-F β-actin-R	CAGCCTTCCT TCTTGGGTATG AGCTCAGTAACAGTCCGCCT
Runx2-F Runx2-R	GGTGAAACTCTTGCCTCGTC AGTCCCAACTTCCTGTGCT
Osterix-F Osterix-R	TGGTACAAGGCAGGCATCCA GGAGCAAAGTCAGATGGGTAAGT
ALP-F ALP-R	ACTGGCTGTGCTCTCCCTAC GACCTCTCCCTTGAGTGTGG
OCN-F OCN-R	CAGGGAGGCAGTGACTCTTC AGTGTGGAAAGTGTGGAGTT
SIRT1-F SIRT1-R	CCAGACCTCCCAGACCCTCAAG GTGACACAGAGACGGCTGGAAC
Beclin-1-F Beclin-1-R	GAATGGAGGGGTCTAAGGCG CCTCTTCCTCCTGGCTCTCT
ATG14-F ATG14-R	CCTCTTCCTCCTGGCTCTCT CTCTTGGTGCCGTTGTGCTCG
LC3-F LC3-R	CTCTCTGAGCCTTAGGTGCC ACTCGTGGGGTGACCATTTC
ATG7-F ATG7-R	CCAGTGACGCCAGATTTCC GGCAGGCACAGATGCTATG

### 2.7 Western blot

After 1 day of stretching, BMSCs were incubated for 30 min on ice in a RIPA lysis buffer (Beyotime, China) to obtain the total protein. The proteins were separated using 8% (for SIRT1) and 15% [for LC3) polyacrylamide gel (Beyotime, China) with an electrophoresis scheme of constant voltage at 70 V for 30 min. Once all the markers had run away, the voltage was changed to 110 V to allow the loading buffer to run to the bottom of the separation gel] and then transferred to PVDF membranes (Bio-Rad System, USA; Millipore, USA, catalog no. ISEQ00010) (membrane transfer scheme: constant current, 250 mA, 90 min). The membranes were then sealed with 5% skimmed milk and incubated with primary antibodies, including actin (1:10,000; Huabio, catalog no. EM21002), LC3 (1:2,500; Proteintech, catalog no. 14,600-1-AP), and SIRT1 (1:1,000; Huabio, catalog no. ET1603-3) at 4°C overnight. After removing the primary antibody, the membranes were then incubated with secondary antibodies (Proteintech, nos. SA00001-1 and SA00001-2) at room temperature for 2 h. The membranes were treated with ECL (Tanon, China, catalog no. 180-501) and exposed to chemiluminescence bands using the Tanon 5200 MuLti automated analysis system. The relative protein expression was calculated using ImageJ (version 1.49v) based on the protein bands.

### 2.8 Alkaline phosphatase staining

The alkaline phosphatase (ALP) activity of BMSCs was assessed through ALP staining after 7 days of mechanical stretching. The cells were treated with 4% paraformaldehyde and then gently rinsed with Phosphate-Buffered Saline (PBS). They were then stained with ALP stain (Sigma) and kept in the dark at 37°C for 20 to 30 min. The staining reaction was stopped, and the cells were observed and photographed under a microscope (Leica). We then used ImageJ (version 1.49) to analyze the images of the ALP staining results, counting the area of stained cell distribution in the images, which represents the amount of ALP secreted in the BMSCs.

### 2.9 Immunofluorescence staining

BMSCs were fixed with 4% paraformaldehyde after 7 days of mechanical stretching. After washing with PBS, the cells were permeabilized with 0.1% Triton X-100 and then were blocked with 10% goat serum (Solarbio, no. SL038). Primary antibodies for SIRT1 (1:100; Huabio, catalog no. ER130811), LC3 (1:100; ABclonal, catalog no. A17424), and Runt-related transcription factor 2 (Runx2) (1:100; ABclonal, catalog no. A2851) were incubated to detect the relative expression and co-localization of these proteins. Finally, the cells were incubated with secondary antibodies DyLight 488 (1:250; BOSTER, catalog no. BA1045-488) and DyLight 550 (1:250; BOSTER, catalog no. BA1133) and then were photographed using a laser confocal microscope (Zeiss LSM 900). We then used ImageJ (version 1.49) to analyze the fluorescence intensity, which represents the protein expression.

### 2.10 MDC staining

Monodansylcadaverine (MDC) staining solution (Beyotime) was added after one-time mechanical stretching, and the BMSCs were then incubated at 37°C for 30 min in the dark. After washing three times with an assay buffer, the cells were observed and photographed by laser confocal microscopy (Zeiss LSM 900).

### 2.11 Statistical analysis

All data are presented as mean ± SD. Comparisons were made using one-way ANOVA, and the Bonferroni *post-hoc* test was performed to detect differences between every two groups. In addition, the Pearson correlation test was performed to examine the relationship between SIRT1 and LC3, as well as LC3 and Runx2 signals based on IF staining. All the statistical analyses were performed using IBM SPSS Statistics 19.0, and *p* < 0.05 was set as the significant difference.

## 3 Results

### 3.1 Exercise prevents bone loss in aging mice

**Figure 2:**
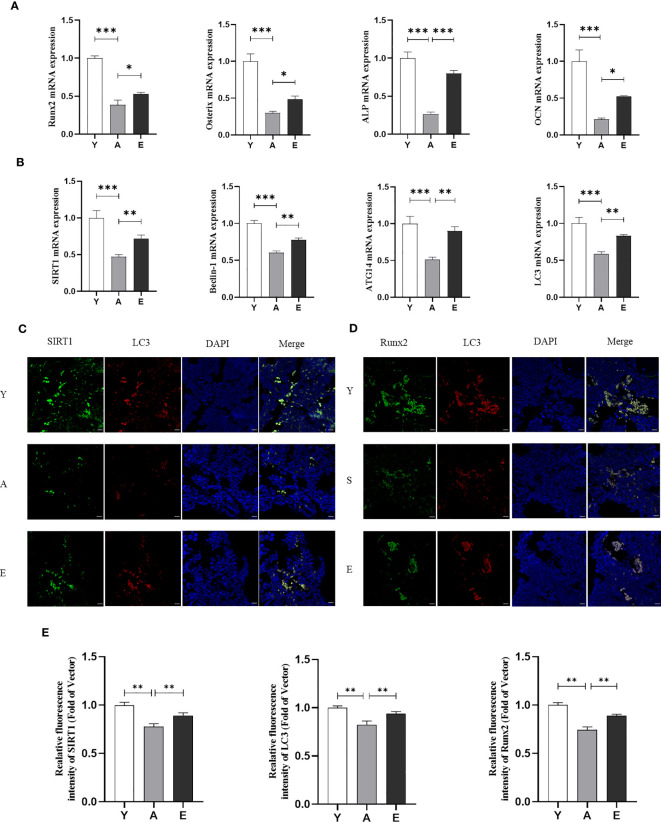
Exercise promotes SIRT1 expression and autophagy levels in aging mice. **(A)** mRNA expression of osteogenic genes inculding Runx2, Osterix, ALP, and OCN in bone tissue. **(B)** mRNA expression of SIRT1 and autophagy genes including Beclin-1, ATG14, and LC3 in bone tissue. **(C)** Representative images of immunofluorescence staining of bone tissue sections to detect the expression and co-localization of SIRT1 and LC3. **(D)** Representative images of immunofluorescence staining of bone tissue sections to detect the expression and co-localization of Runx2 and LC3. Bar, 20 µm; **(E)** Quantitative analysis of fluorescence intensity of SIRT1, LC3, and Runx2. SIRT1, Sirtuin 1; ALP, alkaline phosphatase; OCN, osteocalcin; Runx2, Runt-related transcription factor 2; *P < 0.05, **P < 0.01 and ***P < 0.001. Y, Young control group; A, aging control group; E, exercise group.

To evaluate the effect of treadmill running on the prevention of SOP, we used micro-CT to detect femoral bone mass and structure in the Y, A, and E groups. The results demonstrated that aging mice (A) had a lower bone mass in both trabecular when compared with the young control mice (Y) as Tb.BV/TV, Tb.N, and Tb.Th were significantly decreased in group A, whereas the Tb.Sp was significantly increased. However, 8 weeks of treadmill running exercise significantly increased Tb.BV/TV, Tb.Th, and Tb.N and decreased Tb.Sp in aging mice, whereas no significant difference was detected in cortex bone (**Figures 1B–D**). In addition, the H&E staining also revealed that the A group had significantly lower trabeculae bone than the Y group, whereas the E group improved trabeculae after treadmill running exercise (**Figure 1E**). Furthermore, the expression of osteogenic genes such as Runx2, ALP, Osterix, and osteocalcin (OCN) was lower in the femur of the A group as compared with those in the Y group, whereas those osteogenic genes significantly increased in the E group (**Figure 2A**). These findings indicate that bone mass is reduced accompanied by lower bone formation capacity during aging and exercise promotes bone formation capacity and increases bone mass in aging mice, thereby preventing osteoporosis.

### 3.2 Exercise activates SIRT1 and autophagy in aging mice

We used real-time PCR to detect mRNA expression of autophagy-related factors Beclin1, Autophagy-related protein 14 (Atg14), and LC3 in the bone marrow. The results showed that the expression of these genes was significantly lower in the A group and higher in the E group compared with that in the Y group (**Figure 2B**). Interestingly, it was found that the decrease in autophagy levels in aging mice was accompanied by a reduction in SIRT1 mRNA expression, and exercise activated SIRT1 expression in aging mice (**Figure 2B**). We then performed IF double staining (SIRT1/LC3) on the femurs of mice to investigate the correlation between SIRT1 and autophagy. The results showed that aged bone marrow displayed weaker SIRT1 and LC3 staining compared with young bone marrow, which was reversed by exercise training (**Figures 2C, E**). Similarly, double staining of LC3 and Runx2 in the bone marrow also indicated that LC3 expression accompanied by Runx2 decreased in aging mice (A group compared with Y group) and was reversed after exercise training (E group) (**Figures 2D, E**). Correlation analysis also revealed that SIRT1 was correlated with LC3, and LC3 was correlated with Runx2 (**Supplementary Figure 1**). These findings suggest that the SIRT1–autophagy axis may be involved in aging-related low bone formation and osteogenesis, which is likely to be an important mechanism for improving aging bone health in response to exercise.

### 3.3 Mechanical stretch enhances osteogenic differentiation in BMSCs via SIRT1-mediated autophagy

**Figure 3:**
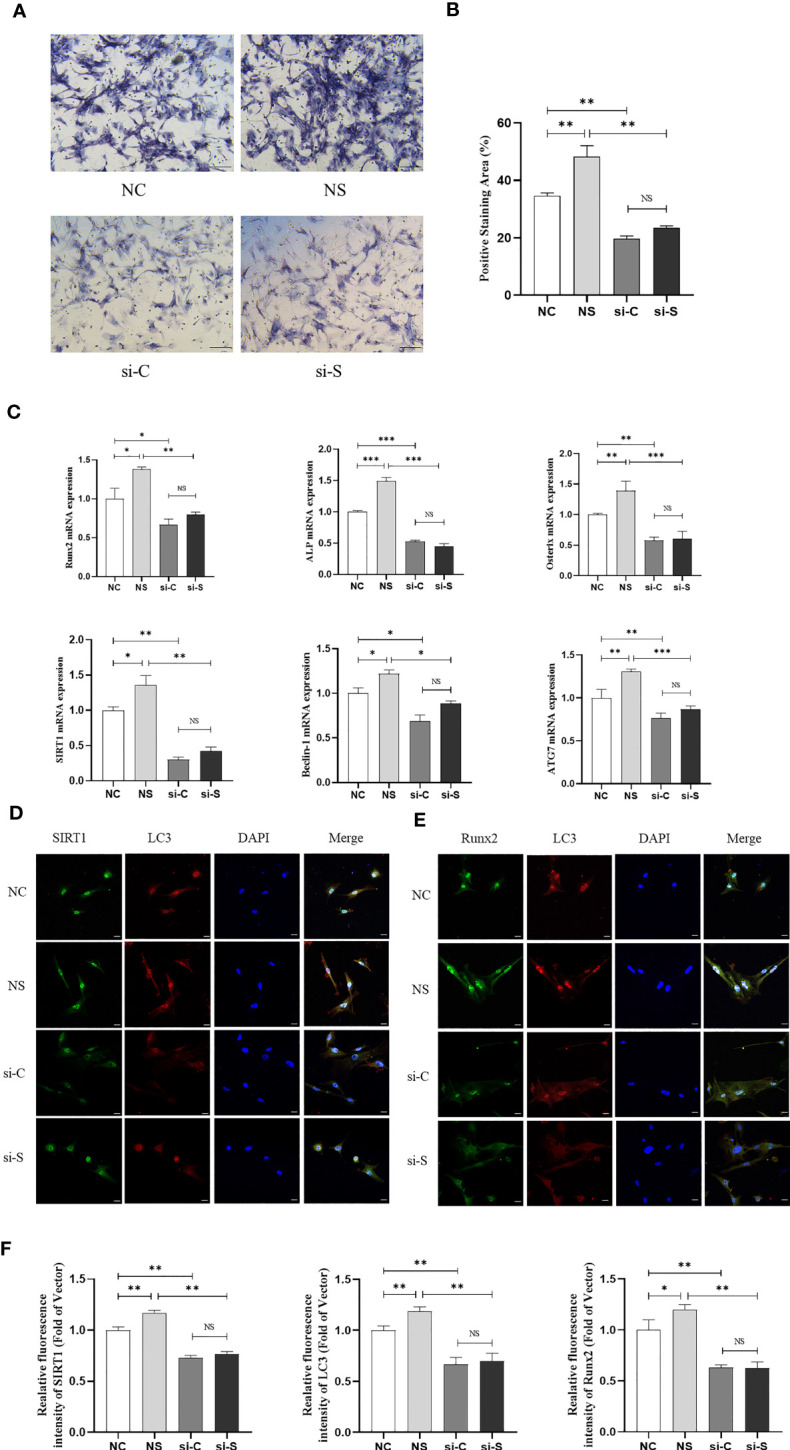
Mechanical stretch promotes osteogenic differentiation and autophagy in BMSCs via SIRT1. SIRT1 was knocked down with siRNA (200 pmol, 72 h) in BMSCs, which were subjected to mechanical stretch every 2 days and harvested after 7 days (the experiments were repeated at least three times). **(A)** Representative images of ALP staining after 7-day mechanical stretch. Scale bar, 100 µm. **(B)** Quantitative analysis of ALP positive staining area. **(C)** mRNA expression of osteogenic genes including Runx2, ALP, and Osterix, SIRT1 and autophagy genes including Beclin-1 and ATG7. **(D)** Representative images of immunofluorescence staining of SIRT1 and LC3 in BMSCs after 7-day mechanical stretch. Scale bar, 20 µm. **(E)** Representative images of immunofluorescence staining of Runx2 and LC3 in BMSCs after 7-day mechanical stretch. Scale bar, 20 µm. **(F)** Quantitative analysis of fluorescence intensity of SIRT1, LC3, and Runx2. ALP, alkaline phosphatase; Runx2, Runt-related transcription factor 2; SIRT1, Sirtuin 1; NC, siRNA-NC; NS, siRNA-NC + mechanical stretch; si-C, siRNA-SIRT1; si-S, siRNA-SIRT1+ mechanical stretch; *P < 0.05, **P < 0.01, and ***P < 0.001; NS, no significant difference.

In the *in vitro* study, BMSCs were isolated and were subject to mechanical stretching interventions. The findings revealed that mechanical loading significantly promoted the ALP staining and mRNA expression of osteogenic genes like Runx2 and ALP in BMSCs. However, knockdown of SIRT1 in BMSCs downregulated ALP staining and those genes expression and then reduced the impact of mechanical stretching on BMSCs osteogenic differentiation (**Figures 3A–C**), suggesting that mechanical stretch promotes osteogenic differentiation in BMSCs by activating SIRT1. In addition, stretch stimulation also promoted the expression of SIRT1 and autophagy-related genes Beclin-1 and ATG7 in BMSCs, whereas SIRT1 knockdown significantly suppressed the expression of SIRT1 and genes related to autophagy (**Figure 3C**). Furthermore, IF staining showed that protein expression of SIRT1, LC3, and Runx2 was significantly increased in BMSCs when subjected to mechanical stretch stimulation, whereas knockdown of SIRT1 was accompanied by a significant decrease in LC3 and Runx2 expression (**Figures 3D–F**). Consistent with the results in mice, a significant correlation was found between SIRT1 and LC3 as well as between SIRT1 and Runx2 (**Supplementary Figure 2**).

**Figure 4:**
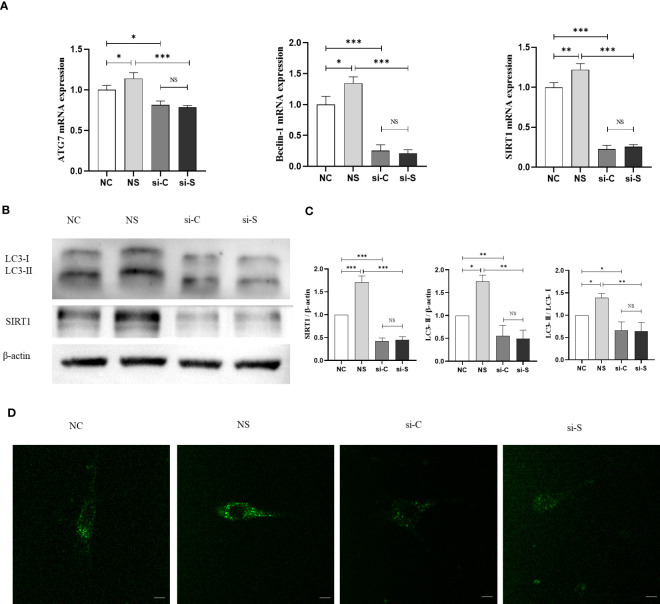
SIRT1 regulates mechanical stretch–induced autophagy in BMSCs. SIRT1 was knocked down with siRNA (200 pmol) in BMSCs, which were subjected to mechanical stretch loading once and harvest after mechanical stimulation (the experiments were repeated at least three times). **(A)** mRNA expression of SIRT1 and autophagy genes including Beclin-1 and ATG7 in BMSCs. **(B)** Representative image of Western blot including the protein of LC3-I, LC3-II, and SIRT1 in BMSCs after mechanical stretch. **(C)** Quantitative analysis of relative protein expression of SIRT1, LC3-II, and LC3-II/LC3-I ratio. **(D)** Representative images of MDC staining in BMSCs after mechanical stretch. Bar, 20 µm. SIRT1, Sirtuin 1; MDC, monodansylcadaverine; NC, siRNA-NC; NS, siRNA-NC + mechanical stretch; si-C, siRNA-SIRT1; si-S, siRNA-SIRT1 + mechanical stretch; *P < 0.05, **P < 0.01, and ***P < 0.001; NS, no significant difference.

To further investigate the mechanism by which mechanical retraction promotes osteogenesis and whether SIRT activates autophagy in BMSCs, we also tested the mRNA levels of Beclin-1 and ATG7 and the shift from LC3-I to LC3-II. The results demonstrated that mechanical stretching significantly upregulated the mRNA and protein expression of SIRT1, accompanied by increased ATG7, Beclin-1, and LC3II. When SIRT1 was knocked down, the expression of ATG7 and Beclin-1 in BMSCs was downregulated, as well as a reduction in the LC3-II/LC3-I ratio, and the effect of stretch stimulation was reduced (**Figures 4A–C**). In addition, MDC staining also showed that stretch stimulation exhibited higher fluorescence density and more autophagic granules, which were inhibited by the knockdown of SIRT1 (**Figure 4D**). These results suggest that mechanical stretch stimulation promotes autophagy in BMSCs by activating SIRT1 expression.

## 4 Discussion

Age-related osteoporosis is characterized by age-related bone loss and specific biological aging in the skeletal system (2). Typically, bone mass in mice peaks at 4–8 months of age and subsequently declines with age. Male C57BL/6J mice showed a 60% reduction in proximal tibial trabecular volume from 6 to 24 months of age, with the most significant decline occurring at 12 months of age (31). In addition, another study showed that male C57BL/6 mice typically exhibit a linear decline in bone density after 42 weeks of age (32). Thus, in this study, 12-month-old naturally aging male C57BL/6J mice were used as a model of SOP. Consistent with the previous studies, the results showed that the BV/TV, Tb.N, and Tb.Th in femoral cancellous bone significantly decreased and the Tb.Sp increased in 14-month-old mice compared with that in 7-month-old mice by the end of the experiment. The H&E staining of longitudinal sections of the distal femur further corroborated the micro-CT results, showing that 12-month-old mice had lower number and volume of trabeculae. These findings suggested that 12-month-old mice were suitable for studying SOP. Studies have demonstrated that treadmill exercise prevents age-related osteoporosis in mice, and our previous study also showed that moderate-intensity running exercise increased bone volume and serum levels of ALP and OCN in senile mice (33–35). Consistent with previous studies, the findings demonstrated that running exercises increased Tb.BV/TV, Tb.N, and Tb.Th of trabecular, suggesting that exercise promotes bone mass and prevents SOP in aging mice, which is also supported by the results of bone histomorphmetry. In addition, the results also revealed that exercise training led to an increase in osteogenic genes such as Runx2, Osterix, ALP, and OCN. These results indicate that exercise promotes bone formation in aging mice, contributing to reduced bone loss with aging.

The pathogenesis of SOP is complicated and involves many factors, of which senescence of BMSCs plays a crucial role. Accumulating evidence has demonstrated that aging accompanied by the osteogenic differentiation potential of BMSCs decreases, and their differentiation into adipocytes increases (7, 36). Promoting osteogenic differentiation of BMSCs or inhibiting adipogenic differentiation is considered a promising developmental strategy against osteoporosis (37). The current study showed that exercise improved the osteogenesis of BMSCs in the bone marrow during senescence.

Autophagy is necessary for maintaining stem cell stemness and differentiation capacity. The progressive decrease in autophagy levels in BMSCs during senescence may contribute to the degenerative changes that occur in senescent BMSCs (38). Increased cellular senescence may be due to age-dependent increases in mitochondrial damage or decreases in autophagy, eliminating dysfunctional mitochondria (39). In the present study, we also found that autophagy levels were significantly decreased in the bone of aging mice as compared with young mice, as evidenced by decreased mRNA levels of Beclin-1, ATG14, and LC3, which was also supported by the reduction of LC3 protein expression in the IF. In addition, running exercise increased autophagy levels in the bone as parallel to the improvement of bone mass in aging mice, indicating that the activation of autophagy levels may be due to the mechanism of exercise improving osteogenic differentiation, which is consistent with other studies. Grumati et al. showed that running exercise enhanced the ratio of LC3-II to LC3-I and autophagosomes in skeletal muscle, which indicated exercise regulated the activation of autophagy (40). Beclin-1 and Atg7 protein expression is upregulated in senile mice following endurance training (41). Chen et al. also found that running exercise reversed the downregulation of Beclin-1 and LC3 II mRNA and protein levels in osteoblasts of T2DM mice, activated autophagy, and promoted bone formation (42).

An important finding of the current study is that exercise improved the expression of mRNA and protein of SIRT1 in the bone, which reversed the reduction of SIRT1 in aging mice. SIRT1 is a histone deacetylase known for regulating various cellular processes such as cell proliferation, apoptosis, autophagy, and senescence (43). SIRT1 not only interacts directly with the ATG family to regulate autophagy but also participates in the regulation of upstream regulators of all steps of autophagy (44). A previous study revealed that the expression of Beclin-1 and LC3 as well as SIRT1 decreased in osteoporotic rats, and further exploration revealed that the reduction of autophagy through SIRT1-mediated activation of p-Akt and p-mTOR may contribute to impaired bone formation capacity during aging (25). This study found that exercise in aging mice improved both autophagy levels in the bone and SIRT1 expression, as evidenced by the results of double IF revealing that SIRT1 increased in parallel with LC3 after exercise training. Moreover, the results of double IF showed that the protein expression of SIRT1 was correlated with LC3 and that LC3 was correlated with Runx2. Therefore, we speculate that exercise activates autophagy via SIRT1, thereby promoting BMSCs differentiation and increasing bone formation.

To further confirm the mechanism, we employed appropriate mechanical stretch stimuli to BMSCs and found that upregulation of the SIRT1 expression in BMSCs during mechanical stretch treatment was accompanied by elevated intracellular autophagy levels and that knockdown of SIIRT1 blocked the activating effect of mechanical stretch on autophagy, thereby reducing the ability of BMSCs to differentiate into osteoblasts. Gu et al. showed that SIRT1 deacetylates nuclear LC3-I at Lysine 49 and Lysine 51. The deacetylated LC3-I interacted with DOR proteins and then the nuclear export of LC3-I to the cytoplasm and interacts with ATG7, which is inserted into the autophagosomal membrane and selectively targets cargo (45). Another study revealed that SIRT1 promotes autophagy by deacetylating FoxO1 and the expression of its target substrates Rab7 and Bnip3, thereby preventing fluoride-induced apoptosis in MC3T3-E1 cells (46). In addition, oxidative stress increases during senescence, and autophagy, as a catabolic process, can remove excessive reactive oxygen species from the body and play a certain antioxidant role (47, 48). Chen et al. demonstrated that mechanical stretch activates SIRT1, induces antioxidant responses in BMSCs, attenuates intracellular reactive oxygen species levels, and improves the osteogenesis of BMSCs (13). Consistent with these studies, this study found that appropriate mechanical stretch promoted the expression of SIRT1 and osteogenesis in BMSCs. The present study extends the findings that SIRT1-mediated autophagy levels in BMSCs may be a curial role in contributing to bone formation. Interestingly, we observed a slight decrease in the molecular of LC3 in the Western blot results. This suggests that SIRT1 may regulate LC3 through phosphorylation, causing a slight alteration in the protein’s molecular weight. Furthermore, combined with the current animal study, we conclude that exercise-induced mechanical stimulation activates BMSCs autophagy and promotes osteogenic differentiation of BMSCs to reduce bone loss during aging and thereby prevent SOP.

### 4.1 Limitations

This study had some limitations. First, this study did not use specific knockout SIRT1 mice to better understand the role of SIRT1 *in vivo*. Second, whether the exercise-mediated SIRT1–autophagy pathway or SIRT1 removes reactive oxygen species from BMSCs in the bone of aging mice was not investigated. Further studies are needed to validated evidence that exercise regulates autophagy and oxidative stress and promotes the potential osteogenic differentiation of BMSCs, thereby improving bone formation and preventing SOP during aging. We also acknowledge that another limitation was the use of only 4-week-old mice to isolate BMSCs, instead of using 12-month-old mice. Nevertheless, given the reduced activity and differentiation ability of BMSCs from aged mice, we deemed using young mice more appropriate for evaluating the mechanical effects on BMSCs. In future studies, we will explore the mechanical loading on BMSCs from aging mice.

## 5 Conclusion

**Figure 5:**
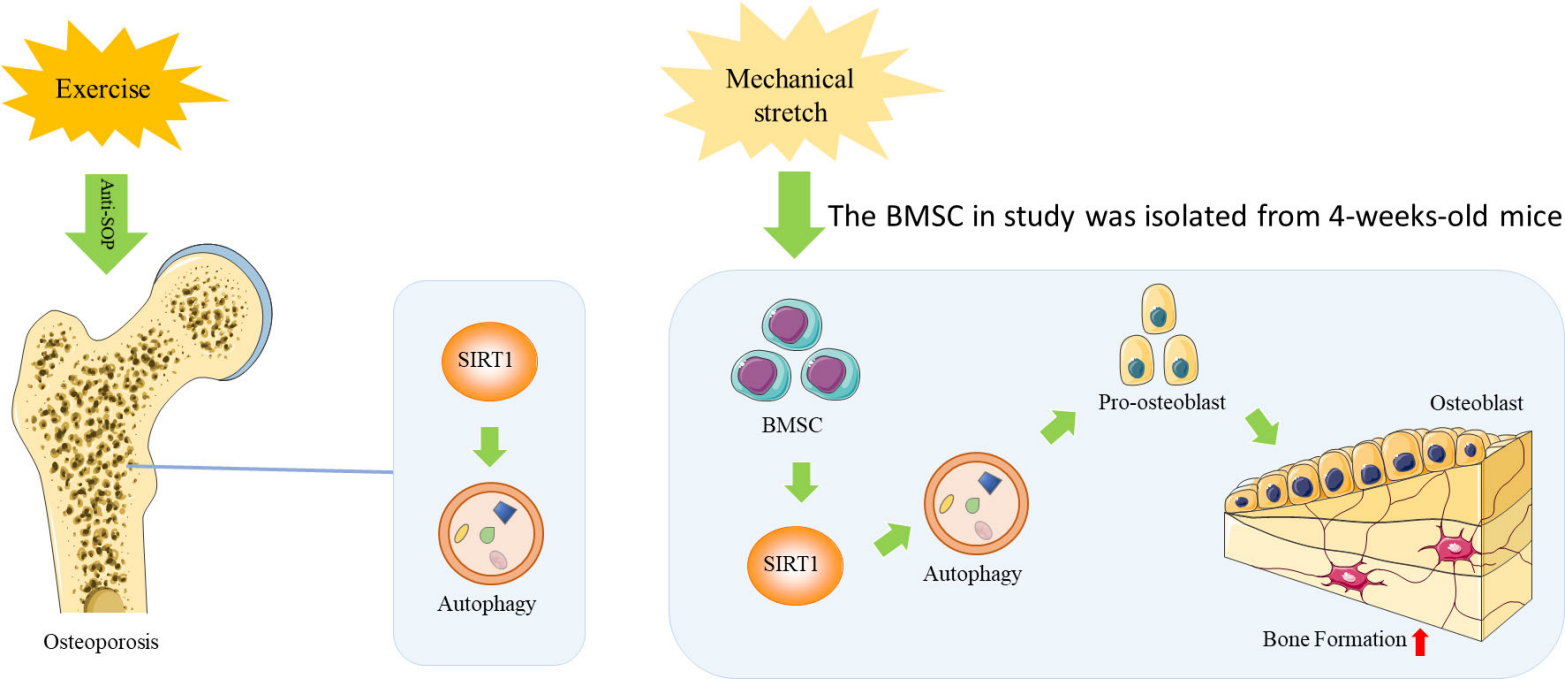
A Schematic diagram of the mechanism by which exercise improves bone health during aging by activating SIRT1-mediated autophagy in BMSCs.

In summary, the findings of this study suggest that exercise improves bone health during aging by activating bone formation, which can be attributed to osteogenic differentiation of BMSCs through the activation of SIRT1-mediated autophagy. The mechanisms underlying this effect may involve mechanical loading. (**Figure 5**). Thus, appropriate exercise and targeting of SIRT1 to enhance autophagy may be a potential therapeutic strategy to attenuate age-related osteoporosis.

## Data availability statement

The original contributions presented in the study are included in the article/**Supplementary Material**. Further inquiries can be directed to the corresponding author.

## Ethics statement

The animal study was reviewed and approved by the Ethical Committee of the Shanghai University of Sport.

## Author contributions

XC, YY, and JZ designed and supervised the whole program. CZ, HD, LS, and XG performed the experiments; CZ, HD, SZ, MH, and XT analyzed the data and prepared the figures. XC, CZ, and LL wrote the manuscript. CZ and XC revised the manuscript. All authors contributed to the article and approved the submitted version.

## Supplementary material

Supplementary Figure 1 Fluorescence intensity-based correlation analysis of SIRT1, LC3, and Runx2 in bone tissue sections and BMSCs. **(A)** Correlation analysis between SIRT1 and LC3, LC3 and Runx2Bone tissue sections; **(B)** Correlation analysis between SIRT1 and DAPI, LC3 and DAPI, and Runx2 and DAPI. BMSCs after mechanical loading.

Supplementary Figure 2 Fluorescence intensity-based correlation analysis of SIRT1, LC3, and Runx2 in BMSCs after mechanical loading. **(A)** Correlation analysis between SIRT1 and LC3, LC3 and Runx2; **(B)** Correlation analysis between SIRT1 and DAPI, LC3 and DAPI, and Runx2 and DAPI.
