# Supplementary figures and images for: Exercise improved bone health in aging mice: a role of SIRT1 in regulating autophagy and osteogenic differentiation of BMSCs
Source: Front Endocrinol (Lausanne). 2023 Jul 4;14:1156637. doi: 10.3389/fendo.2023.1156637 (PMC10355118; doi:10.3389/fendo.2023.1156637)

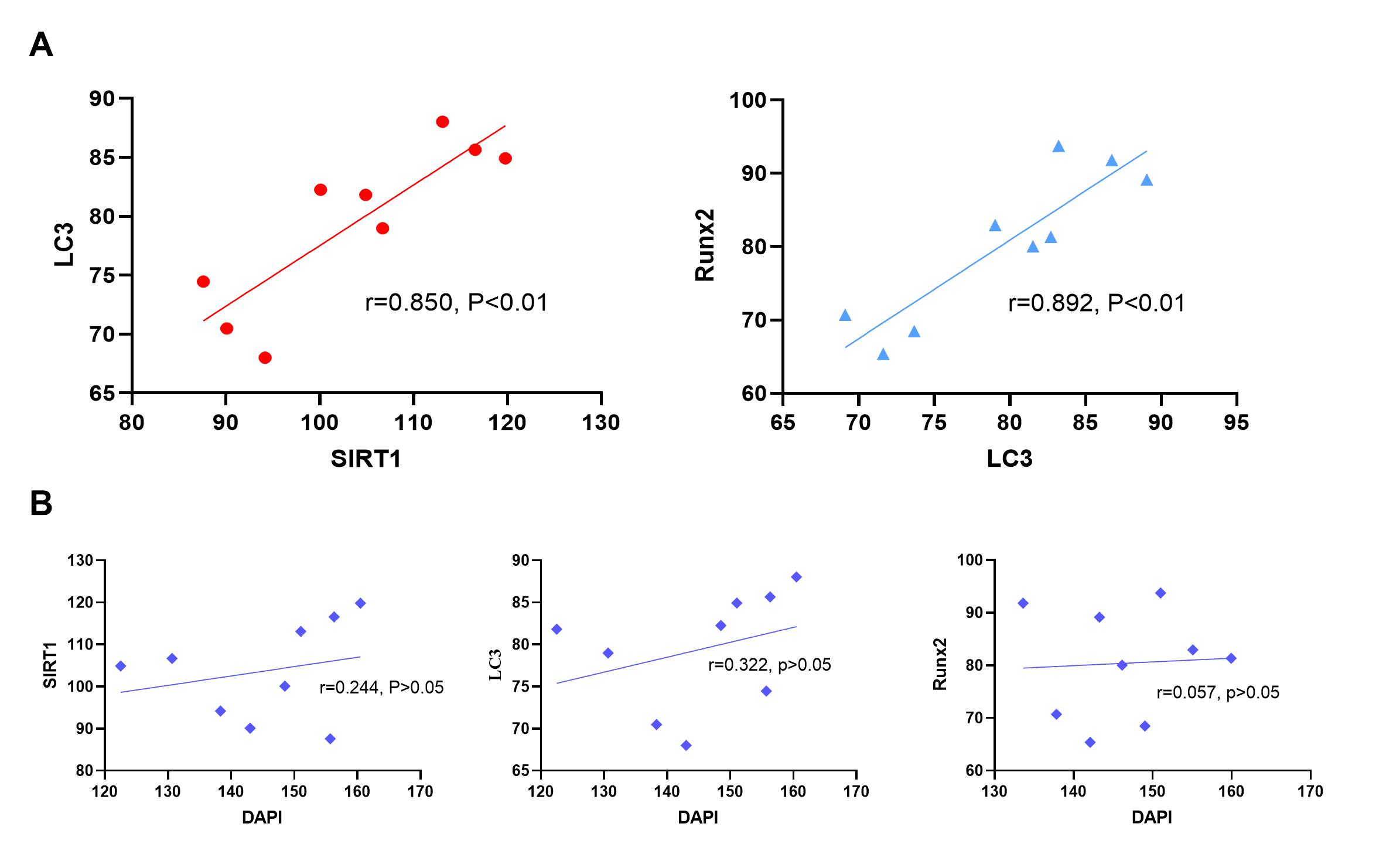

Supplement: Supplementary Figure 1 — Fluorescence intensity-based correlation analysis of SIRT1, LC3, and Runx2 in bone tissue sections and BMSCs. (A) Correlation analysis between SIRT1 and LC3, LC3 and Runx2Bone tissue sections; (B) Correlation analysis between SIRT1 and DAPI, LC3 and DAPI, and Runx2 and DAPI. BMSCs after mechanical loading. [file Image_1.jpeg]

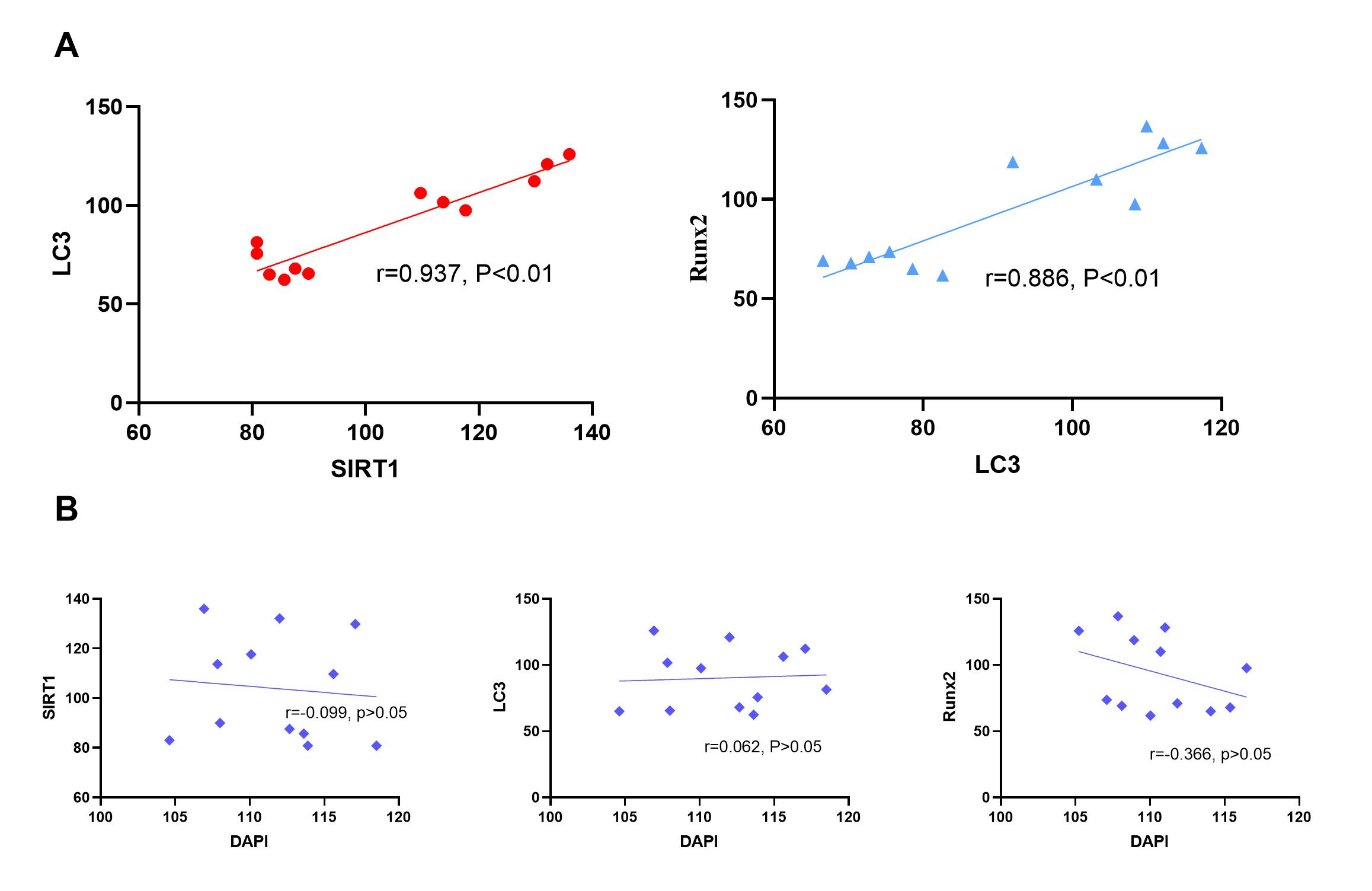

Supplement: Supplementary Figure 2 — Fluorescence intensity-based correlation analysis of SIRT1, LC3, and Runx2 in BMSCs after mechanical loading. (A) Correlation analysis between SIRT1 and LC3, LC3 and Runx2; (B) Correlation analysis between SIRT1 and DAPI, LC3 and DAPI, and Runx2 and DAPI. [file Image_2.jpeg]
